# Microbiota-derived short-chain fatty acids: Implications for cardiovascular and metabolic disease

**DOI:** 10.3389/fcvm.2022.900381

**Published:** 2022-08-11

**Authors:** Yingdong Lu, Yang Zhang, Xin Zhao, Chang Shang, Mi Xiang, Li Li, Xiangning Cui

**Affiliations:** ^1^Department of Cardiology, Guang’anmen Hospital, China Academy of Chinese Medical Sciences, Beijing, China; ^2^First Clinical Medical School, Shandong University of Traditional Chinese Medicine, Jinan, China

**Keywords:** short-chain fatty acids, CVD, inflammation, glucolipid metabolism, blood pressure, gut-brain axis

## Abstract

Cardiovascular diseases (CVDs) have been on the rise around the globe in the past few decades despite the existing guidelines for prevention and treatment. Short-chain fatty acids (SCFAs) are the main metabolites of certain colonic anaerobic bacterial fermentation in the gastrointestinal tract and have been found to be the key metabolites in the host of CVDs. Accumulating evidence suggest that the end-products of SCFAs (including acetate, propionate, and butyrate) interact with CVDs through maintaining intestinal integrity, anti-inflammation, modulating glucolipid metabolism, blood pressure, and activating gut-brain axis. Recent advances suggest a promising way to prevent and treat CVDs by controlling SCFAs. Hence, this review tends to summarize the functional roles carried out by SCFAs that are reported in CVDs studies. This review also highlights several novel therapeutic interventions for SCFAs to prevent and treat CVDs.

## Introduction

In the past 20 years, the incidence of cardiovascular diseases (CVDs) has increased significantly, and brings staggering health and economic burden. Stroke and ischemic heart disease were the main causes of death in China in 2017 (1, 2). Multiple pathological factors influence the initiation and development of CVDs, including atherosclerosis (AS), hypertension, myocardial infarction, heart failure (HF), stroke, and arrhythmia (3–6).

In current years, the gut and its microbiota have been identified as crucial factors in the development of CVDs (7–10). Several bacterial factors, such as the metabolite trimethylamine oxide (TMAO), tryptophan metabolites, and endotoxin, were demonstrated to affect CVD development (11–13). Changes in gut microbiota (GM) and its constituents, gene abundance, and specific species or flora can affect TMAO. GM disorder can increase TMAO level in the body, thus accelerating the aging of vascular endothelial cells (ECs), affecting the immune system, promoting inflammatory response, and causing the occurrence or aggravation of vascular diseases (14–16). In addition, GM changes also lead to oxidative stress response, sodium metabolism, low density lipoprotein oxidation, and affect the progression of vascular diseases (17, 18).

In short-chain fatty acids (SCFAs), the fermentation products of intestinal microorganisms, especially acetate, propionate, and butyrate were confirmed to reduce intestinal PH value, inhibit pathogenic microorganisms, and maintain intestinal barrier function (19–23). The decease of SCFAs production *in vivo* could lead to the inhibition of the G protein-coupled receptor (GPCR) pathway and the increase of the expression of inflammatory factors, such as INF-γ, leading to lipid metabolism disorder, aggravation of inflammation and vascular remodeling, acceleration of arterial thrombosis, and ultimately the occurrence and aggravation of AS, hypertension, pulmonary arterial hypertension, cerebrovascular diseases, and other diseases (24–27). Due to the development of next-generation sequencing (NGS) technique, metabolomics and bioinformatics analysis, the relationship between SCFAs and CVD has moved from previous associative studies to those that elucidate the cause-effect.

In this review, we gave an overview of gut-derived SCFAs, including acetate, propionate, and butyrate production, transport, and signal transduction and their associations with CVDs. Then, we reviewed the mechanisms of regulating the pathological process, and discussed the role of SCFAs targeted therapy in the progress of CVDs.

## The production of short-chain fatty acids and signal transduction

### Short-chain fatty acids substrate and biosynthesis

Short-chain fatty acids, major produced by the specific intestinal microbiome in cecum and colon, mainly include acetate, propionate, and butyrate (constitute > 95% of the whole SCFAs) with approximate molar ratio of 3:1:1 in intestinal lumen (28). Indigestible saccharides, such as dietary fibers, none starch polysaccharides (NSP), or resistant starch (RS), that escape digestion in the small bowel are highly anaerobic glycolysis and generate SCFA in the colon (29). Intriguingly, the amino acids from proteolytic are alternative substrate for SCFAs biosynthesis when the routine fibers are in short supply (30). Moreover, to a lesser extent, the minority SCFAs (formate, valerate, and caproate, which make up the remaining < 5%) can be fermented by chain amino acids, such as leucine, valine, and isoleucine (31). The microbiome converting the fermented fibers to major end production is mediated by complex enzymatic pathways. Although more studies are needed to verify the exact commensal microbes producing SCFAs, much information about what kinds of taxa responsible for which metabolites yield is available (Table 1).

**TABLE 1:** Short-chain fatty acids (SCFA) production, absorption and receptors.

SCFAs	Major metabolic location	Synthetic route	Producers	References
Acetate	Colon, kidneys, sympathetic nervous system, blood vessels, enteroendocrine L cells, the vasculature, immune cells	Acetyl-CoA pathway	Enteric bacteria, e.g., *Akkermansia muciniphila, Bacteroides* spp., *Bifidobacterium* spp., *Prevotella*spp., *Ruminococcus* spp.	Brandsma et al. (148); Battson et al. (149)
		Wood-Ljungdahl pathway	Acetogenic bacteria	
Propionate	Colon, kidneys, sympathetic nervous system, blood vessels, enteroendocrine L cells, the vasculature, immune cells	Succinate pathway	*Bacteroides* spp., *Phascolarctobacterium succinatutens*, *Dialister* spp., *Veillonella* spp., several Firmicutes, and Bacteroidetes	Brandsma et al. (148); Colman and Rubin et al. (150)
		Acrylate pathway	*Megasphaera elsdenii* and *Coprococcus catus*, a few members of the families Veillonellaceae and Lachnospiraceae	
		Propanediol pathway	*Salmonella* spp., *Roseburiainul inivorans*, *Ruminococcus obeum, Proteobacteria* and members of the Lachnospiraceae family	
Butyrate	Colon, kidneys, sympathetic nervous system and blood vessels, enteroendocrine L cells, the vasculature and immune cells,	Phosphotransbutyrylase/ butyrate kinase route	*Coprococcus comes*, *Coprococcus eutactus*	Donohoe et al. (38); Brandsma et al. (148)
		Butyryl-CoA: acetate CoA-transferase route	*Coprococcus comes*, *Coprococcus catus*, *Coprococcus eutactus*, *Anaerostipes* spp., *Eubacterium hallii*	

### Short-chain fatty acids absorption

The absorption of SCFAs is efficient and rapid with varying concentration along the whole length of the gut *via* putative mechanism: the monocarboxylate transporter 1 (MCT-1) and the sodium-coupled monocarboxylate transporter 1 (SMCT-1) receptors (32, 33). The highest SCFAs level is in the cecum and proximal colon at concentrations between 10 and 100 mM as the energy sources for colonic epithelial cells to maintain the intestinal integrity or as a signal molecular (34, 35). Although a majority of SCFAs are metabolized in the colon, a small percentage is absorbed in peripheral blood with the concentration of 19–160 μmoL/L for acetate, 1–13 μmoL/L for propionate, and 1–12 μmoL/L for butyrate (34).

### Signal transduction

Besides serving as intestinal fuel (butyrate) or nutrition for colonic mucosa (36, 37), with the development of human diseases, the function of SCFAs also existed in intestinal epithelial cells, immune cells, and adipocytes. Two major mechanisms might be involved. One is as the histone deacetylases inhibitor (HDACi) to connect with the transcriptional machinery. Butyrate is identified as the most potent HDACi activity of the three, followed closely by propionate performing anti-cancer and anti-inflammatory response (38, 39). The other mechanism is coupled with GPCRs, including GPR41 (propionate > butyrate > acetate), GPR43 (propionate = butyrate = acetate), and GPR109A (only respond butyrate). GPCRs are expressed in intestinal epithelial cell, adipocytes, neurons, immune cells, or even vascular endothelium. Although the mechanism has not been fully revealed between SCFAs and associated receptors, increasing studies have highlighted the beneficial effects of SCFAs on CVDs and we will discuss in the next sections.

## Short-chain fatty acids in cardiovascular diseases

**FIGURE 1:**
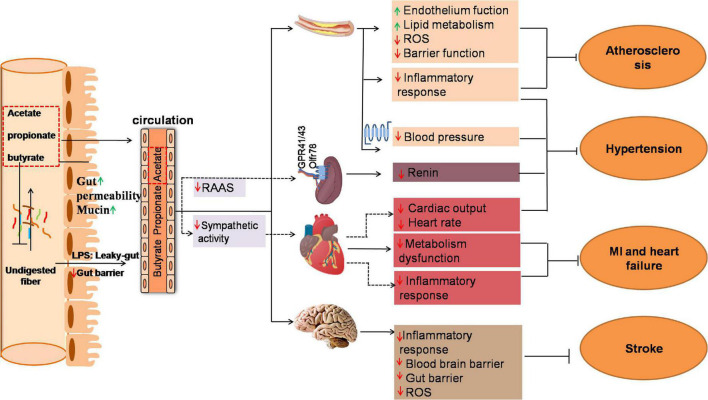
Mechanism of gut microbiota produced SCFAs in several key tissues under cardiovascular disease (CVD). Fermentation of undigested fiber in the distal intestine leads to the production of SCFAs. The ration of acetate to propionate to butyrate in the ileum, cecum and colon is ∼ 3:1:1. The change of gut microbial diversity, abundance and functions called “dysbiosis” due to overnutrition increase intestinal permeability which allows intestinal luminal endotoxin to translocate to systemic circulation through the “leaky-gut.” The elevated endotoxin in vascular, heart and brain lead to “low-grade” inflammation response and ultimately exacerbate CVD such as atherosclerosis, hypertension, myocardial infarction, heart failure and stroke. Propionate and butyrate are generally metabolized in the colon that mainly affect local gut. SCFAs in distal colon could effectively alleviate gut barrier dysfunction by modulating intestinal permeability and secretion of mucus. In addition, small amount of propionate and butyrate and major acetate are absorbed into circulation and modulate the function of coronary artery, kidney, heart, and brain which might effectively improve CVD. Solid lines indicate direct SCFA effects and dashed lines indicate indirect SCFA effects. GPR, G-protein coupled receptor; MI, myocardial infarction; RAAS, Renin-angiotensin-aldosterone System; ROS, Reactive oxygen species.

With the aid of the genomics and other omics tools, science researchers have uncovered the impact of SCFAs on cardiac pathogenesis (Figure 1).

### Atherosclerosis

**FIGURE 2:**
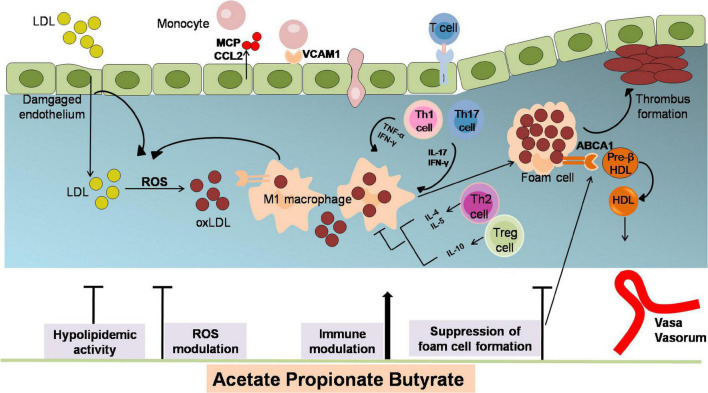
The anti-atherosclerotic effects by which SCFAs alleviates the development of AS. Atherogenesis begins with the adhesion of blood leukocytes to the activated endothelial monolayer. Activated endothelial cells express leukocyte adhesion molecules such as VCAMs that capture blood monocytes. The captured monocytes (the most numerous of the leukocytes recruited) matures into macrophages, uptake oxLDL (LDL oxidized by ROS), promote foam cells formation and ultimately yield plaque lesion. Moreover, T cells are observed to differentiate into various subsets of T cells and establish their important modulatory roles. Among them, T helper (TH)–1 and TH17 cells are atherogenic via producing pro-inflammatory cytokines [interferon-γ (IFN-γ)] and activating macrophages, while regulatory T (Treg)cells specific for oxLDL inhibit lesion formation and progression via generating IL-10 and TGF-β. The role of SCFAs in inhibiting atherosclerosis includes attenuating lipid profile and ROS, reducing monocyte adhesion, cholesterol aggregation, macrophage inflammation and foam cells formation. ABCA1, ATP binding cassette A1; CCL2, chemokine (C-C motif) ligand 2; HDL, high density lipoprotein; IFN-γ, interferon-γ; LDL, low density lipoprotein; MCP, chemotaxis protein-1; oxLDL, oxidized low-density lipoprotein; TNFα, tumor necrosis factor; Th, T helper cells; Treg, regulatory T cells; VCAM1, vascular cell adhesion molecule-1.

Atherosclerosis is a chronic disease of the arterial wall, which is related to myocardial infarction and stroke (40–43). The underlying pathophysiological mechanisms of AS are lipid deposition, inflammatory response, oxidative stress damage, and endothelial dysfunction (40, 41, 43–45). SCFAs have been proved critical in modulating AS pathological process (Figure 2).

#### Hypolipidemic activity and suppression of foam cell formation

Dyslipidemia level is correlated with the risk of AS and its complications in human populations. In the environment of hyperlipidemia, lipids can be processed into to a mixture of oxidation products and proteins (oxLDL), producing foam cells and stimulating the progression of AS. SCFAs play important roles in lipid metabolism. Studies found that butyrate inhibited the absorption of intestinal cholesterol and promoted the excretion of cholesterol in intestinal cells by regulating the expression of mRNA-associated transporter, which significantly ameliorated AS induced by apoE^–/–^ mice diet (46). It was also found that butyrate pretreatment reduced the atherosclerotic plaque area of mouse aortic arch by 50%, decreased the absorption of oxLDL (oxidized low-density lipoprotein), and reduced the formation and deposition of foam cells in the plaque (47). Other studies have made in-depth research on SCFAs and AS from the perspective of genes. Du uncovered that butyrate lowered the level of several lipogenic genes, such as acyl-CoA thioesterase1(Acot1), Acot2, Perilipin 2 (Plin2) and Plin5 and fatty acid degradation-associated genes including Cyp4a10, Cyp4a14, and Cyp4a (21). Butyrate could induce the transcription of fibroblast growth factor 21 (FGF21) by inhibiting HDAC3 in diet obese mice, thus promoting lipid oxidation, triglyceride clearance, and ketogenesis in the liver (48).

Foam cell formation, which is characterized by accumulation of oxLDL in macrophages, is a sign of early AS, and it binds to scavenger receptors (SR), such as CD 36 and oxLDL (49). ATP binding cassette A1 (ABCA1) can suppress macrophage transformation and contribute to the attenuation of AS (50). Previous studies have demonstrated that butyrate supplementation decreases CD36 expression in peritoneal macrophages from ApoE^–/–^ mice stimulated by oxLDL (51). It has been reported that butyrate accelerates cholesterol efflux by activating the expression of Sp1/ABCA1 pathway, which promotes the reuse of cholesterol in liver macrophages or its final elimination in ApoE^–/–^mice. This is that butyrate regulates cholesterol catabolism by up-regulating biosynthesis of bile acid synthesis rate-limiting enzymes (21).

#### Short-chain fatty acids modulate immune cell and inflammation

Atherosclerosis is closely associated with chronic vascular inflammation, and the overproduction of inflammatory cytokines and the expression of adhesion molecules are the two important segments in the development of AS. The intensified inflammation of the artery wall can lead to the instability of atherosclerotic plaque and the formation of occlusive thrombus, thus leading to atherosclerotic CVDs events. The beneficial effects of SCFA by modulating the systemic inflammatory response to slow down AS are well established. Inflammatory signaling in vascular endothelium stimulates the biosynthesis of various effector proteins, including endothelial-leukocyte adhesion molecule-1 (*E*-selectin), vascular cell adhesion molecule-1 (VCAM-1), and chemokines, such as IL-8 and chemotaxis protein-1 (MCP-1), which promotes recruitment and retention of circulation monocytes to the injured endothelial monolayer. Therefore, reducing inflammatory transmitters is an important step in preventing the development of AS. Study has reported that SCFA inhibits the production of proinflammatory cytokines through the activation of GPR41 and GPR43, and butyrate promotes preservation of endothelial function *via* attenuating inflammatory factor and thereby exert anti-atherosclerotic action (52). Previous studies have revealed that the intrinsic mechanism of acetic acid on IL-6 and IL-8 is due to signal transduction mediated by GPR 41/43, while the effects of butyrate and propionate on IL-8 production and VACM-1 expression were mediated by HDAC 11. Experiments *in vitro* confirmed that butyrate decreases the production of VCAM1 and chemokine (C-C motif) ligand 2 (CCL2) in ECs upon stimulation of TNFα (47). Similarly, study also uncovered that SCFAs influence LPS- or TNFα-induced endothelial activation by inhibiting the production of IL-6 and IL-8, and reducing the expression of VCAM-1 and subsequent cell adhesion (51). Besides, butyrate has been proved to decrease the release of MCP1/CCL2 in human ECs stimulated by oxLDL and hence decrease the migration monocytes to the lesion area.

After that, monocytes differentiate into macrophages, which aggravated AS by transforming into foam cells and secreting a large number of pro-inflammatory factors. Macrophages express different markers under the induction of different cytokines, and thus differentiate into different subtypes, including M1 and M2 macrophages. Proinflammatory cytokines, such as IL-1, IL-6, IL-8, and TNF-α, are released by M1 macrophages *via* nuclear factor κB (NF-κB) pathway after lipid uptake, and ultimately stimulate foam cells yielding. Conversely, anti-inflammatory (IL-10) cytokines and growth factors (TGF-β) secreted by M2 inhibit the progression of AS (53). Makoto has shown that butyrate and propionate attenuating-α production through blocking NF-κB pathway activation by lipopolysaccharide (LPS) in human peripheral blood mononuclear cells. Butyrate and propionate can rescue ApoA-I transcription in human liver cells under inflammatory conditions by peroxidase-activating receptor (PPAR)-mediated trans-activation inhibition of NF-κB (54). Moreover, butyrate has been shown to increase IL-10 production and decrease TNF-α simulated expression of MCP-1 and VCAM in plaque lesion, and the inner mechanism is also associated with the suppression of NF-κB pathway in ApoE^–/–^ mice (51). Kasahara reported that tributyrin (TB) can inhibit the development of atherosclerotic lesions in germ-free ApoE^–/–^ mice model partly due to the decrement of aortic inflammation indicated by decreasing the relative mRNA levels of TNF-α and VCAM1 in aortic root (55). The study also found that intestinal administration of butyrate reduced endotoxemia and the development of AS and indicated that intervention measures aimed at increasing the representation of butyrate-producing bacteria may provide protection against AS (55). SCFA can also exert its anti-inflammatory effects by interfering with the activation of key signaling proteins, including the MAPK protein ERK (extracellular regulated protein kinase), and can exert its anti-atherosclerotic effects by inhibiting systemic inflammation (56). In terms of the pathological mechanisms of butyrate-modulated inflammation, Kasahara unraveled that butyrate significantly lowered gut permeability, as well as plasma LPS levels. Previous study reported that SCFAs could directly promote T-cell differentiation and produce interleukin-17(IL-17), IFN-γ, and/or IL-10 depending on HDAC inhibitor activity in different cytokine milieu (57). SCFA has different effects on the activation of the endothelial NLRP3 inflammasomes and the associated neointimal formation of the arterial wall. Butyrate may have beneficial effects on vascular inflammation or AS by inhibiting the production of superoxide anions and activation of NLRP3 inflammasomes (52).

Butyrate and propionate have been proved to stimulate the extrathymic generation of Treg cells (58). Unexpectedly, although SCFAs have been found to induce the differentiation of regulatory T cells, butyrate supplementation did not affect levels of CD4^+^T cells and Treg cells in spleen or para-aortic lymph node samples in AS models (55). Moreover, B2 cells, one of the mature B cells, are TH2-cell-dependent and promote AS by producing specific IgG antibodies against its homologous antigen (such as oxLDL). The anti-oxLDL/oxLDL immune complex is an inflammatory signal that triggers macrophage activation (59). In terms of the effects of SCFAs on the complex, previous evidence has uncovered that butyrate is capable of decreasing contents of IgG anti-oxLDL and attenuating remitting inflammatory response (60). Haghikia revealed a new immune-mediated pathway connecting propionate with intestinal Npc1L1 (Niemann-Pick C1-like 1) expression and cholesterol homeostasis. The findings emphasize the gut immune system as a potential therapeutic target to control dyslipidemia that be a promising strategy for prevention of atherosclerotic CVDs (61).

#### Inhibition of oxidative stress

Under oxidative stress situation, NO reacts with superoxide to form peroxynitrite anion. The inactivation of NO contributes to the inflammatory and thereby exerts pro-atherosclerotic action (62, 63). Classical markers of oxidative stress are the superoxide ion (O^2–^) and nitro tyrosine, whose local and systemic levels are positively correlated with CVDs (64). NADPH oxidase is one of the major enzymes involved in ROS production and has been positively correlated with the progression of AS (65). Aguila demonstrated that the reduction of oxidative stress was related to the lower production by NADPH oxidase (60). *In vitro* studies, butyrate, can reduce the production of reactive oxygen species (ROS). Butyrate pre-treatment of endothelium and peritoneal macrophages yielded a lower oxLDL-stimulated production of superoxide and hydrogen peroxide. Aguilar also demonstrated that oral administration of butyrate positively influences plaque composition by decreasing nitro tyrosine and induces NO synthase (iNOS) formation in lesion site of ApoE^–/–^ mice (60). Previous pieces of evidence have uncovered butyrate treatment that reduces the levels of ROS in vascular smooth muscle cells (VSMCs) by upregulating the level of glutathione-S-transferase (GST) (66). Investigations suggest butyrate is attributed to a blockade of lipid raft redox signaling platforms to produce O2•- upon 7-Ket or CHC stimulations to prevent AS (52). Stamm demonstrated that different oxidants have different vasodilating capacities for the endothelium-dependent vasodilator acetylcholine (ACh) *in vitro* compared to inorganic nitrite. They elucidated oxidants react with NO released by eNOS after acetylcholine stimulation to form the intermediate peroxynitrite, thereby reducing the potency of this endothelium-dependent vasodilator (67).

Taken together, SCFAs are crucial in anti-inflammation, anti-oxidation, and lipid modulation in the progression of AS. SCFAs have been developed as a beneficial microbial product with favorable anti-atherosclerotic effects and are potential therapeutic target for the treatment of AS.

### Hypertension

Systemic arterial hypertension is an independent risk factor for CVD (68, 69). Accumulating clinical cohorts have proved SCFAs as an important factor to regulate blood pressure. One cohort included 54 males (38 hypertensive, 7 borderlines, and 9 normotensive) claiming that the stool levels of SCFAs in hypertensive are higher than normotensive individuals based on 24-h ambulatory BP measurements (70). On the contrary, other cohorts have uncovered that the deficient SCFAs production due to long-term low fiber westernized diets increase the prevalence of hypertension. Supporting these results, a cross-sectional study consisted by 29 non-treated hypertensive and 32 normotensive subjects presented a positive correlation between the feces level of SCFAs and systolic and diastolic blood pressure (71). Similarly, in another SPRING study (the Study of Probiotics in Gestational Diabetes) constituted by 205 obese pregnant women at 16 weeks showed that systolic and diastolic blood pressure was associated with altered GM composition and butyrate production. Therefore, they concluded that increasing butyrate producing capacity may be a new perspective for the maintenance of normal blood pressure in obese pregnant women (22). Bartholomeus has suggested that lifestyle modifications leading to augmented SCFAs production could be beneficial for hypertensive CVDs patients (20).

#### Modulation the classic blood pressure regulatory modes

Chronic activation of the sympathetic nervous system (SNS) and rennin-angiotensin-aldosterone system (RAAS) contributes to hypertension by excessive production of catecholamines, such as noradrenaline and adrenaline, angiotensin II, and aldosterone, which stimulate adrenoceptors, angiotensin II receptor type 1 (AT1), and mineralocorticoid receptors to increase vascular tone, renal sodium and water reabsorption and heart rate. In an angiotensin II (Ang II)-induced rat hypertension model, intramedullary infusion of sodium butyrate lowered mean arterial pressure by suppressing renal receptor (PRR)-mediated intrarenal rennin-angiotensin system indicated by the renal expression of PRR, angiotensinogen, angiotensin I-converting enzyme and rennin (72). In a rodent study, propionate has been demonstrated to modulate rennin release combined with Olfr78 receptor which located in the renal juxtaglomerular apparatus to mediate hypertensive response (73). Similarly, acetate supplementation in hypertension mice downregulated the rennin angiotensin system in the kidney and attenuated the blood pressure. Moreover, high salt consumption aggravates the kidney burden which has consistently been implicated in hypertension. Across over, trial has found that modest sodium reduction increases circulating SCFAs in untreated hypertensives (74). Furthermore, SNS receives signals from brain regions, such as forebrain, hypothalamus, and brainstem, and has projections to all major blood pressure-regulating organs, including the heart, blood vessels, and kidneys. Study has uncovered that propionate may modulate the SNS activity at the level of sympathetic ganglion *via* GPR41 and decrease the heart rate, which is closely related with the cardiac output and blood pressure (75). Researchers found that the hypotensive effect of butyrate may result from decreasing sympathetic activity *via* colon vagus nerve signaling, which depended on the afferent colonic vagus nerve signaling independent of SCFA receptor (76).

#### Inflammatory response modulation

Elevated levels of circulating cytokines and C-reactive protein (CRP) marked hypertension as a low-grade inflammatory disease involving innate and adaptive immune responses (77, 78). The tissue injury resulted from non-immune mechanisms of hypertension leads to DAMP formation, such as ROS, LPS, and high mobility group box 1 (HMGB1). Within the innate immune systems, macrophages and DCs detect DAMPs and thus produce pro-inflammatory cytokines and chemokines accumulating in vasculature and kidney. Within the adaptive immune system, effector T cells and B cells in the help of DCs can directly influence renal tubular sodium transport and vascular resistance through pro-inflammatory cytokines releasing. All of these promote blood pressure elevation, while the immunoregulatory pathways involving Treg cells have salubrious effects on BP by producing IL-10. In a study of 441 community-dwelling adults, Cuesta-Zuluaga claimed that individuals with higher butyrate excretion have fewer lipopolysaccharide-binding protein (LBP) level and is associated with hypertension (79). In addition, Ganesh have uncovered that acetate supplementation could prevent OSA-induced gut inflammation and hypertension in obstructive sleep apnea (OSA)-induced rat hypertension model (80). Similarly, in terms of acetate against hypertension, another DOCA-salt hypertensive mice model study has shown that fiber consumption with high acetate concentration lowered IL-1 signaling, an early response pro-inflammatory cytokine originated from macrophages. They also found that acetate downregulated the gene for early growth response protein 1 (Egr 1) mRNA involved in cardiac hypertrophy, cardiorenal fibrosis, and inflammation according to cardiac and renal transcriptome analysis (19). Moreover, propionate has been shown to mitigate systemic inflammation responses quantified as a decrement of splenic effector memory T cell, Th17 cells, and increment of Treg cells in AngII-induced hypertensive model. Bartolomaeus uncovered that the beneficial effects of propionate in AngII-infused hypertensive mice are Treg-dependent as this kind of effect was abrogated in Treg-depleted Ang II-infused mice (20).

#### G protein coupled receptor regulation

Emerging evidence implicate that SCFAs are associated with reduced blood pressure and less incidence of cardiovascular mortality (66–68). Mortensenet firstly uncovered that SCFAs can dilate isolated human colonic resistance arteries in 1990 (81). SCFA played important parts in rising blood pressure by Olfr (73). Oflr78 and its human ortholog (OR51E2) as a novel SCFA receptor (specifically acetate and propionate) localize in the afferent arteriole part of the juxtaglomerular apparatus (JGA) where they mediate the rennin secretion. The mainly described function of Olfr78 is related to glomerular filtration and blood pressure regulation effect (73, 82). Propionate could combine with Oflr78, elevate cytosolic cAMP, promote renin releasing and exert a chronic hypertensive effect when it entered the circulation *via* colonic absorption (81, 83, 84). Conversely, the effect of propionate on renin release was absent in Olfr78-/-mice65. Pluznick found that propionate administration caused a large, rapid drop in blood pressure in Olfr78-deficient mice, while this hypotensive also manifestation in wide-type mice indicating antagonized effect on Olfr78 exists on BP. This team ultimately has revealed that GPR41co-expression with Olfr78 in the smooth muscle cells of resistance vessels has a hypotensive response on vascular (73). GPR41 appears to induce vasorelaxation with the help of vascular endothelium (85). Consistent with this, oral administration of propionate took a hypertensive effect in Gpr41 null mice (86). GPR41 and Olfr78 likely played opposing roles in the regulation of blood pressure. An antibiotic treatment experiment on wide type and Ofr78-deficient mice highlighted the dual roles of SCFA-mediating hypotensive effects combined with Gpr41 and hypertensive effects antagonized by Oflr78, pressure was significantly increased in Oflr78-deficient mice the mean blood. Altogether, these kinds of opposing responses may produce a “buffering” effect to defense the wide swings in blood pressure due to the normal, physiological variations of SCFAs.

### Myocardial infarction

Myocardial infarction (MI) will occur when coronary artery stenosis reaches more than 80%, downstream myocardial cells are ischemic, and oxygen demand is increased under high workload (87, 88). SCFAs were associated with the levels of local and system inflammation, oxidative stress, apoptosis, and metabolism regulation in MI pathogenesis.

#### Modulation of inflammatory response

**FIGURE 3:**
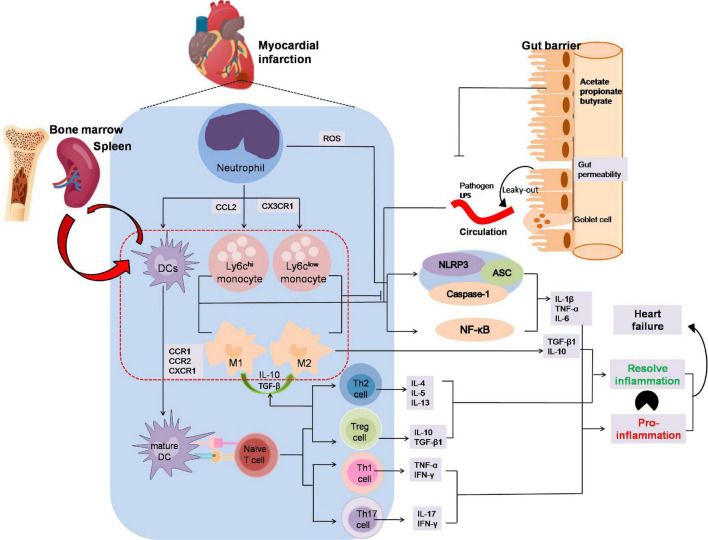
The anti-inflammation effects by which SCFAs alleviates the development of myocardial infarction. Neutrophils are the first immune cells to infiltrate infracted myocardium and then activated by adhesion and chemokines released from injured endothelial and ROS from injured endothelium after MI. Ly6c*^hi^* monocytes captured by CCL2 are recruited early to engulf necrotic debris and apoptotic myocardial cells mediated by NF-κB or the NLRP3 pathway in the first phase, and anti-inflammatory Ly6c*^low^* monocytes dependent on CX3CR1 occur later to participate in myocardial repair by releasing anti-inflammatory factor. DCs activated by CCR1, CCR2 or CXCR1 present antigen to naïve T cell. Th1 and Th17, two of the CD4^+^ subsets, enhance IFN-γ, TNFα and IL-17 secretion, whereas Th2 and regulatory T cells reacting to activate IL-10 or TGF-βto increase M2 macrophages polarization and dampen inflammation. In addition, the leak-gut effect of dysbiosis may exacerbate the pro-inflammation effect on MI. The imbalance between inflammatory phagocytosis and anti-inflammatory response would aggravate MI. In MI progression, SCFAs might promote M2 macrophages polarization to suppress inflammatory responses and inhibit macrophages cells secreting the pro-inflammatory factor and avoid aggravation of viable areas of the myocardium. Moreover, SCFAs play an important role in T cells polarization and activation, especially Treg cells which could alleviate the inflammation injury in MI progression. ASC, apoptosis-associated speck-like protein containing CARD; CCR, chemokine (C-C motif) receptor; CXCR3, chemokine C-X3-C-Motif Receptor 1; DC, dendritic cell; NLRP3, nucleotide-binding domain leucine-rich repeat proteins 3.

Inflammatory processes play an important role in MI (89, 90). SCFAs have been reported to participate in the recruitment, activation, and polarization of leukocyte after MI and this may open a new avenue to improve forms of immunotherapy for MI patients (Figure 3). Although SCFAs have been demonstrated to influence neutrophil recruitment, inflammatory mediators, effector functions, and apoptosis related to the immune response (91–94), rarely few studies pointed out the mutual influence of SCFAs and neutrophil variation in the development of myocardial infarction.

Short-chain fatty acids have been demonstrated to drive myelopoiesis in the bone marrow and tissue-resident monocyte infiltration. Dietary supplementation with propionate can modulate immune system and subsequent cardiac repair *via* restoring the level of myeloid cells and CX3CR1 + monocytes infiltration to the peri-infarct zone in antibiotic-treated MI mice (95). They also demonstrate that an SCFA-producing probiotic mixture is associated with both increased levels of myeloid cells in the hearts of antibiotic-treated MI mice and enhancement of propionate levels. *In vitro* studies, SCFAs exert anti-inflammatory response by decreasing the production of cytokines, such as IL-10 and prostaglandin E2 (PGE2) in human monocytes and peripheral blood mononuclear cells (PBMC) (96, 97). In some *in vitro* studies, macrophage incubated with SCFAs decreased the LPS-induced TNF-α, IL-1β, IL-6 secretion, and the inner mechanisms are associated with the NF-κB and the NLRP3 pathway (98). An intestinal macrophage study demonstrated that the anti-inflammatory effect of butyrate was mediated by HDAC inhibition (99). Jiang demonstrated that butyrate administration down-regulated the expression of inflammatory cytokines (TNF-α, IL-1β), up-regulated the IL-10 levels in the infarct border zone, and ameliorated cardiac function probably through promoting M2 macrophages polarization to suppress inflammatory responses at 3 days post-MI (100). The mechanisms that orchestrate such divergent functions remain unknown. However, a timely resolution of inflammation by manipulating the M2/M1 ratio might be a strategy to prevent infarct expands and left ventricular dilates. Taken together, SCFAs could not only promote monocytes into the injury site to remove the necrosis tissue in the first phase of MI, but also could inhibit macrophages cells secreting the pro-inflammatory factor and avoid aggravation of viable areas of the myocardium.

There may be a link between MI and adaptive immune cells, CD4^+^T cells deficiency and CD8^+^T cells are associated with worse outcome in MI patients. SCFAs play an important role in T cells polarization and activation, especially Treg cells. Arpaia found that butyrate facilitated extrathymic generation of Treg cell sin mice (58). In addition to butyrate, *de novo* Treg-cell generation in the periphery was potentiated by propionate (57). Bartholomeus established a cause-effect relationship between anti-inflammatory effects of regulatory T cell and cardioprotective effects of propionate (20). They have found that propionate administration decreased spleen effector T cells, Th17 cells, and increased Treg cells which ultimately protected cardiac damage and remodeling. This cardioprotective effects of propionate were abrogated in regulatory T cell-depleted mice (15). In conclusion, SCFAs play vital roles in myocardial infarction *via* immune regulation. Nevertheless, most of the literatures linking SCFAs and immune response in CVD are studied in cells or rodent model, additional work is needed to understand which patient population would benefit from SCFAs production.

#### Metabolism regulation modulation

Deeper insight acquired that SCFAs also impact metabolism, which underlie susceptibility to CVD (101). Only few studies stated its direct effect on myocardial metabolic disorders, most of them are dabbling in maintaining the lipid and glucose homeostasis of obesity and T2DM subjected to metabolic disease. Butyrate, as the most important fuel for the intestine epithelium, could act as an energy source in AMI therapy (102). Cheng found that sodium butyrate injected into ischemic zones in AMI model rat could promote mobilization of cellular energy store and angiogenesis, inhibit ROS generation, and contribute to cardiomyocyte protection by binding to the Sirt3 with the function of NADP + cycle located in the mitochondria (103). Based on these findings, butyrate may be a new therapeutic agent following AMI as one type of nutrient. Zhang has demonstrated that sodium butyrate protected against HFD-induced cardiac ventricular dysfunction and metabolic disorders in T2DM model compared with the HFD-fed mice. They found that butyrate could attenuate metabolic dysfunction by activating p38/PRAK pathway which has been demonstrated to promote the GLP-1 receptor-induced protective effect (63). In another study, propionate-treated modified cardio-protection through improving mitochondrial anomalies which played a central role in energy metabolism control coupled with GPR41 (104). Further study is warranted to elucidate the directly causal effect of metabolism and function between SCFAs and heart both in animal studies and human trial.

### Heart failure

Atherosclerosis, hypertension, and other comorbidities such as obesity and T2DM typically impede infarct healing, cause hypertrophy, fibrosis, and subsequently lead to HF. Experiments *in vivo* confirmed that sodium butyrate forcefully attenuates Ang II-induced rat cardiac hypertrophy as indicated by decreased ratio of heart weight/body weight and cardiomyocyte size, attenuated extensive fibrosis, and inflammation, and the inner mechanism is due to repressing the activation of COX2/PGE2 pathway in an HDAC-dependent manner (105). Similarly, Umadevi showed that butyrate reduces natriuretic peptide receptor-A (NPRA) gene (Npr1) copies-elicited cardiac hypertrophic, interstitial fibrosis, and inflammation by inhibiting HDAC1 and HDAC2 (106). Furthermore, elevated cardiac fibrosis and left ventricular hypertrophy are normalized in the presence of acetate supplementation, which is attributed to downregulate the mitogen-activated protein kinases (MAPK) signaling in the heart (19).

Several studies implied that imbalanced composition and function of gut microbiome, known as dysbiosis, increases the risk of incident adverse cardiovascular events, including HF (107–109). The current “gut hypothesis of HF” firstly proposed by Tang implied that decreased cardiac output and adaptive redistribution of systemic circulation led to intestinal hypoperfusion, intestinal villi ischemia, bowel wall edema, and impaired barrier function. This disruption in intestinal barrier function in turn leads to the increment of gut permeability and circulating endotoxins (LPS), augment inflammatory-related respond and escalated HF (110–112). It is worth mentioning that SCFAs play an important role in maintaining intestinal barrier and regulating immune response in HF progression. Clinical trials have shown that patients with HF have a thickened intestinal wall, colon, suggesting intestinal edema, and increased collagen accumulation in mucosal of the small intestine. A441 community-dwelling adult study found that fecal SCFAs was negatively correlated with the serum lipopolysaccharide-binding protein (LBP) concentrations, a biomarker produced to response to LPS microbial translocation and a marker of gut permeability (79). Moreover, butyrate has been reported to increase mucus production and tight-junction protein expression, such as zonulin and occluding, contributing to the decrement of intestinal permeability in a GPR43-dependent manner (113). New mechanistic insight surrounding the impact of SCFAs on gut barrier integrity and cardiomyocyte function is provided *via* inhibiting NLRP3 inflammasome and autophagy (114). Further study is warranted to elucidate the direct effect of SCFAs on gut barrier and cardiac function in HF.

### Stroke

Similar to myocardial infarction, cerebral ischemic stroke is caused by focal occlusion or arterial stenosis, as well which leads to the interruption of cerebral blood supply and consequently brain dysfunction (115). Recently, the “brain-gut axis,” a bidirectional communication system between the brain and the gut and its microbiota has been demonstrated as a hot area in stroke. Increasing evidence demonstrated that the SCFAs appear to be the most likely missing link along the gut-brain axis and might be able to modulate stroke and post-stroke recovery. Yamashiro found a 13% decrease in total organic acids with SCFAs accompanied by increased abundance of several genera and species correlated with the inflammation independent of age, T2DM, and hypertension in patient with stoke (116). Another study aimed to explore the association between post-stroke cognitive impairment (PSCI) implied that SCFAs could predict 3 months or longer PSCI early and accurately after stroke onset (117). Consistently, SCFAs supplementation is found to be effective treatments for stroke by controlling barrier structure, metabolism, inflammation, and GM dysbiosis.

#### Interfere with the gut microbiota

Cerebral ischemia causes GM dysbiosis, which may initiate a cascade of events, including increasing intestinal permeability which allows the translocation of GM to systemic circulation through the “leaky-out” that deteriorates the outcome of stroke. The GM itself or metabolites directly or indirectly mediate neural communication and maturation. Remodeling the GM may offer an effective treatment for stroke. In a middle cerebral artery occlusion (MCAO) stroke model, the dysfunction of GM and decreased acetate, propionate, and butyrate occurred in ischemic stroke. Supplementation with butyrate significantly increased the α-diversity of the GM in cerebral ischemic stroke and effectively relieved stroke (118). In addition, butyrate treatment could alter the gut microbial composition of rats with cerebral ischemic stroke enriched the populations of more beneficial bacteria, such as lactobacillus, butyric coccus, and megamonas (118).

#### Inflammatory response attenuation

Stroke alters the GM composition, and in turn, microbiota dysbiosis has a substantial impact on stroke outcome by modulating the immune response. Microglial cells are the brain’s resident immune cells against a variety of external and internal insults of neurodegenerative diseases, stroke, and traumatic brain injuries. SCFAs have been studied to modulate the post-stroke neuronal plasticity mediated by circulating lymphocytes on microglial activation (119). Brain transcriptomic analysis indicated that microglia was the main cellular target of SCFAs on the effect of synaptic, and the inner mechanism is associated with NF-κB pathway (119). Sodium butyrate has a strong anti-inflammatory against LPS-induced responses as indicated by the decreasing levels of TNF-α, NOS2, Stat1, and IL-6 both in rat primary microglia and MCAO model (119). Sodium butyrate decreased the number of monocytes/macrophages *via* inhibiting HDAC activity in permanent middle cerebral artery occlusion (pMCAO) model (120). Taken together, SCFAs may primarily affect lymphocytes already in the peripheral immune compartments which then secondarily mediate microglial changes in the cerebral immune milieu after brain invasion.

#### Reactive oxygen species and apoptosis

Activated microglial and invading leukocytes exert a cytotoxic function by releasing ROS and apoptotic protein which lead to brain infarction and excitotoxicity. COX-2 catalyzes the production of proteinoid and free radicals. NO produced by iNOS after focal cerebral ischemia regulates the activity of COX-2 (121). Sodium butyrate has been elucidated to inhibit NO and COX-2 expression to attenuate the ischemic injury. Furthermore, HSP70, a critical effect against apoptotic and cell death, and Bcl-2, a typical anti-apoptotic protein, were super induction in ischemic brain by post-insult sodium butyrate due to HDAC inhibition in pMCAO model (120). Butyrate-produced bacteria was demonstrated to improve neurological deficit scores by decreasing the expression of caspase-3, Bax and increasing the ratio of Bcl-2/Bax. In addition, clostridium butyricum pretreatment attenuates cerebral ischemia/reperfusion injury in mice *via* anti-oxidations indicated by the decrement of MDA and the increment of SOD (122).

#### Blood-brain barrier

After stroke, the integrity of the blood-brain barrier (BBB) is an important consideration for brain protection. Several tight junction proteins between ECs, the major component of BBB, prevent the paracellular diffusion of various molecules in the blood to the brain and its extracellular fluid. Matrix metalloproteinases (MMPs) mediate BBB disruption and vasogenic edema after cerebral ischemia by degrading the extracellular matrix, basal lamina proteins, and tight junctions around the BBB (123). Sodium butyrate can attenuate BBB disruption *via* decreasing BBB permeability and MMP9 activity in the dependent of HDAC activity in transient focal ischemia model (123). In another study, germ-free adult mice non-colonized with clostridium tyrobutyricum (produces mainly butyrate) or bacteroides the taiotaomicron (produces mainly acetate and propionate) enhanced BBB integrity as indicated by the increment of the tight junction proteins occluding and claudin-5. In addition, gavage of sodium butyrate in germ-free adult mice gains the same effect.

Thus, microbiota-produced SCFAs treatments are a promising strategy for stroke, while further studies are needed to illuminate the cause-effect between SCFAs on BBB and stroke.

### Arrhythmia

Cardiac arrhythmias are the abnormalities or perturbations in the normal activation or beating of heart myocardium. Few researches have studied the SCFAs on the cardiac arrhythmias. One study showed that butyrate could improve ventricular arrhythmia (VA) following MI *via* shorten P wave duration and QJ intervals. Particularly, butyrate could inhibit sympathetic neural remodeling *via* decreasing the density of nerve fibers for growth-associated protein-43 (GAP-43) and tyrosine hydroxylase (100). Study also demonstrated that propionate reduced susceptibility to VAs in hypertensive rat models. Besides, propionate improved the electric remodeling confirmed by attenuated cardiac gap junction remodeling and lateralization of connexin 43 in cardiomyocytes. The effect of SCFAs on cardiac arrhythmias should be further studied in animal and human studies (20).

## Therapeutic intervention

Cardiovascular disease is accompanied by the existence of many risk factors, such as obesity, T2DM, hypertension interacted with multi-organs, and tissues showing lipid and glucose metabolism disorders, oxidative stress, systemic inflammation, and other performance. Although conventional treatment of CVDs has played a role in clinical practice, many links between the SCFAs and susceptibility for CVD have placed SCFAs as a novel target for therapeutic in the future. Several approaches to manipulate the SCFAs hold promise, including diet regulation, fecal microbiota transplantation, prebiotics, probiotics, and traditional Chinese medicine (TCM).

### Dietary interventions

Dietary modulation of nutritional interventions is an effective strategy for CVD prevention and therapy. The World Health Organization stated that the daily consumption of grains, as well as 400 g per day of fresh fruits and vegetables, is recommended in daily intake of fiber. A meta-analysis consisted by 22 cohort studies reveal that dietary rich in fiber is inversely associated with risk of CVD (124), and higher intake fiber may contribute to lower bold pressure in patients with hypertension (125). Mice fed with high fiber diet or supplemented with acetate present a higher level of acetate-producing bacteria, which prevent the development of hypertension and HF (19). In addition, Mediterranean diet characterized by large quantities of fruit, vegetables, cereals, legumes, olive oil, moderate quantities of fish, poultry, and dairy products and low quantities of red meat and wine contribute to high SCFAs levels or SCFAs-producing bacteria, which can consequently modulate CVD pathogenesis. A systematic review examined dietary patterns, such as dietary approaches to prevent hypertension and other diets that delay HF progression. The authors analyzed that the adoption of Mediterranean or the dietary approaches to stop hypertension (DASH-type) diet patterns showed a protective effect on the incidence of HF and/or worsen the cardiac function parameters (126). Similarly, a randomized intervention trial concluded that men and women who consumed a Mediterranean diet for 6 months could improve vascular endothelial function and blood pressure (126). One study specifically demonstrated that habitually following high-level adherence to a Mediterranean diet could significantly increase the levels of fecal SCFAs (127). Another study found that the subject’s adherence to the Mediterranean owned a positive correlation with intestinal total SCFAs. Moreover, an increase of 10% of SCFAs accompanied with decreasing inflammatory cytokines, such as VEGF, MCP-1, IL-17, IP-10, and IL-12, after a 3-month low-calorie Mediterranean diet on people had a low-to-moderate cardiovascular risk profile (128). Taken together, consumption of a high fiber diet would be a promising intervention to reduce CVDs risk, and one of the inner mechanisms may be associated with the level of SCFAs.

### Traditional Chinese medicine

The TCM has multiple components and targets for the treatment of CVDs, which has a synergistic therapeutic efficacy. In recent years, many studies proved that the active ingredients of Chinese medicine as a therapeutic intervention to treat CVD through interacting with microbial metabolites have been reported.

Studies showed that TCM has gradually become important role in the progression of metabolic syndrome by regulating the intestinal flora SCFAs level. Studies found a variety of TCM polysaccharides that could regulate the production of SCFAs to maintain blood glucose homeostasis in diabetic model. Nie found that the polysaccharide purified and isolated from *Plantago asiatica* L. (PLP) could significantly increase the concentration of SCFAs and decrease the glycemia, lipid profile in high-fat diet-induced T2DM rats, which speculate that the anti-diabetic effect of PLP in T2DM rats may be related with the augmented levels of SCFA (129). Hydroxysafflor yellow A (HYSA) promoted the SCFAs-produced bacteria and increased the production of acetate, propionate, and butyrate, which improve insulin resistance and glucose tolerance (130). Study found that concentration of propionate is negatively correlated with hyperglycemia on high-fat diet-induced T2DM mice with high-dose of total saponins intervention (131). Nie also found that the increasing SCFAs level by PLP was accompanied with decreasing plasma lipid markers, such as total cholesterol and triglycerides, because of propionate inhibiting cholesterol synthesis in rat hepatocyte. Other studies showed that ethanol extract of Ganoderma lucidum and Xiexin Decoction have the potential to reduce serum TG, TC, and LDL-C levels (132–134).

Zhou found that the high-salt diet-induced ejection fraction-preserving rat HF (HFpEF) model treated with Xiao-Qing-Long Tang (XQLT) had a lower Firmicutes/Bacteroidetes ratio, higher acetate, propionate and butyrate concentration compared with the HF group (135). They speculated that the prevention of the HFpEF development by XQLT may be associated with decreased inflammatory cytokines *via* SCFAs (135). In addition, other studies found that simulated gastric juice, simulated intestinal fluid, and human fecal flora incubated with S-3-1 extracted from Sijunzi decoction could regulate the inflammatory-related flora and increase the content of acetate and total SCFAs (136). Other Chinese medicines, such as berberine and Kyolic aged garlic extract, have been proved to inhibit the expression of pro-inflammatory genes, such as TNF-α, IL-1β, IL-6, inducible nitric oxide synthase, and cyclooxygenase-2 (137, 138).

Traditional Chinese medicine has important role in intestinal barrier integrity. Baicalin is commonly used to treat inflammatory bowel diseases (IBD) and hypertension. Wu found the abundance of SCFAs-producing bacteria increased after baicalin interfered with hypertensive rats, the concentration of intestinal metabolites SCFAs increased and the concentration of butyrate was positively correlated with the expression of tight junction protein (139). It is speculated that baicalin can maintain intestinal integrity and reduce systemic inflammation by up-regulating the production of SCFAs. Garlic extract has probiotic properties which increase the richness and diversity of intestinal microorganisms by increasing the production of intestinal mucus, especially stimulating the growth of lactobacillus and clostridium to reduce blood pressure (139).

Taken together, the TCM could function as metabolic, inflammation, and gut barrier regulator which may hold promise for the prevention or treatment of CVD. We need to ask rigorous mechanism between the TCM, SCFAs, and the CVD.

### Probiotics and prebiotics

Probiotics are defined as live beneficial bacteria which are administered to re-establish an appropriate intestinal balance. Another strategy for modulating intestinal microbiota is the use of prebiotics, which are non-microbial entities provided to elicit a favorable impact on microbial community composition and function. Probiotics and prebiotics may potentially act through different mechanisms, including gut permeability, lipid metabolism, and blood pressure with pathogens.

Experiments uncovered probiotics and prebiotic may be potential therapeutic intervention for cardiovascular disorder by modulating the SCFAs level. In an animal study, RS deficiency contributed to a series of pathological alterations in C57BL/6J mice, including reducing acetate-producing microbiome and up-regulating blood pressure (140). In a human clinical trial, stable coronary artery disease patients supplemented with prebiotic was reported to increase plasma propionate level, improve endothelium-dependent vasodilation and systemic inflammation (141). Moreover, daily body restore (DBR), a mixture of nine probiotics organisms of the genera *Lactobacillus* and *Bifidobacterium* and 10 digestive enzymes, was supplemented in mice model of hypercholesterolemia, the level of propionate increased and transverse colon and reversed the lipid profile associated with AS (142). In another study, administration of 14 probiotics in db/db mice generated anti-diabetic effect, which was associated with the increment of propionate, butyrate, and SCFA-producing microbial community levels. Probiotics administration also effectively improves the function of intestinal barrier, insulin resistance. All these results indicate that probiotics might be an effective way to prevent diabetes progression (143). It was found that not all humans respond to dietary changes in a similar manner, and non-responsiveness to either a fiber-rich or weight loss diet was shown to correlate with pre-intervention increased bacterial diversity.

In conclusion, these studies elucidated that individualized treatment programs based on microbiome and SCFAs may provide novel treatment strategies for CVD. Further research to explore the potential mechanism and adverse effects of probiotics and prebiotics in treating CVD is warranted.

### Fecal microbiota transplantation

Gut microbial modulation by fecal microbiota transplantation (FMT) is a possible therapeutic intervention designed to displace intestinal pathogens by introducing fecal contents from healthy subjects into the gastrointestinal tract of patients. Some of FMT alter the gut microbiome composition thus increasing the production of certain SCFAs. In an animal study, the Dahl salt-sensitive (S) mice transplanted with cecal contents of Dahl salt-resistant (R) mice had significantly higher systolic blood pressure and mean blood pressure than those of S mice transplanted with autologous (144). In another study, Ldlr^–/–^mice were transplanted with fecal intestinal flora of Caspase1^–/–^ (Casp1^–/–^) mice, and Ldlr^–/–^ mice transplanted with autologous fecal flora of Ldlr^–/–^ mice were served as control group. After 13 weeks of high-fat cholesterol-rich feeding, Ldlr^–/–^ (Casp1^–/–^) mice showed larger atherosclerotic lesion size in the aortic root, higher level of inflammation, and lower cecal concentrations of propionate, acetate, and butyrate compared with Ldlr^–/–^ (Ldlr^–/–^) mice (145). Similarly, in a human clinical study, 18 obese patients with metabolic syndrome infused with a microbiome solution from lean healthy males in small intestine showed a 2.5-fold increase in the number of butyrate production intestinal bacteria in their stool, and an increase of insulin sensitivity compared to the group infused with an autologous gut microbiome solution (146). Microbiota transplantation may play crucial roles in modulating the composition of intestinal flora, regulating blood pressure, increasing insulin sensitivity, reducing inflammation, and arteriosclerosis. A meta-analysis revealed that fecal microbial transplantation is safe as a therapeutic intervention of inflammatory bowel diseases. Whether adverse reactions will occur after MT may be related to the disease being treated and the patient’s physical condition (147).

Generally, the application of microbiota transplantation requires caution and more deeper studies are needed on dosing, delivery route, and formulation for intestinal flora transplantation.

## Conclusion

Multiple animal and human clinical studies have suggested an important link between intestinal microbial metabolism SCFAs and CVDs. Several pivotal mechanisms might be responsible for the putative positive effects of SCFA on CVDs pathogenesis.

The regulation of intestinal barrier to prevent the pathogens or bacterial endotoxins into the system and modulate the inflammatory response on the immune and periapical tissue by SCFAs takes important roles in the myocardial infarction or HF progression, the plaques formation in AS, blood pressure control, and insulin resistance of T2DM. In addition, the capacity of SCFAs ensuring the energy homeostasis and lipid buffering capacity *via* metabolic regulation and gut-brain axis participating take a crucial part during CVDs progression. It is worthy to note that each SCFA-driven mechanism pathway does not exist independent and better understanding of them would greatly facilitate managing cardiac health especially preventing CVDs. Focusing on the SCFAs manufacturing, diet, TCM, probiotics and prebiotics, and fecal transplantation offer some novel potential therapeutic opportunities for CVDs.

Furthermore, despite these exciting and intriguing findings, few studies have provided casual evidence of a direct participatory role of SCFA and poor intervention therapeutic studies gave an explicit and direct relationship with SCFAs to the development of CVDs. A better understanding of SCFA-host and intervention measures-SCFAs-host are needed in future studies.

## Author contributions

XC conceived the idea, critically reviewed, and proofread the manuscript. YL performed the literature search, drafted the manuscript, and drew the figures. YZ assisted in drafting and editing. CS assisted with instructive layout of figures. LL and XC gave constructive comments. All authors read and approved the final manuscript version.
